# Disease characteristics and clinical specific survival prediction of spinal ependymoma: a genetic and population-based study

**DOI:** 10.3389/fneur.2024.1454061

**Published:** 2024-09-13

**Authors:** Tengyue Fu, Chuxiao Mao, Zhuming Chen, Yuxiang Huang, Houlin Li, Chunhua Wang, Jie Liu, Shenyu Li, Famu Lin

**Affiliations:** ^1^Guangdong-Hong Kong-Macau Institute of CNS Regeneration (GHMICR), Jinan University, Guangzhou, China; ^2^The Department of Neurosurgery, The First Affiliated Hospital of Jinan University, Guangzhou, China; ^3^College of Life Science and Technology, Mudanjiang Normal University, Mudanjiang, China; ^4^The Department of Neurosurgery, The Second Affiliated Hospital of Guilin Medical University, Guilin, China; ^5^The Department of Neurosurgery, Shunde Hospital of Southern Medical University, Foshan, China

**Keywords:** spinal ependymoma, *CELF4*, SEER, nomogram, prognosis

## Abstract

**Background:**

Spinal Ependymoma (SP-EP) is the most commonly occurring tumor affecting the spinal cord. Prompt diagnosis and treatment can significantly enhance prognostic outcomes for patients. In this study, we conducted a comprehensive analysis of RNA sequencing data, along with associated clinical information, from patients diagnosed with SP-EP. The aim was to identify key genes that are characteristic of the disease and develop a survival-related nomogram.

**Methods:**

We first accessed the Gene Expression Integrated Database (GEO) to acquire the microarray dataset pertaining to SP-EP. This dataset was then processed to identify differentially expressed genes (DEGs) between SP-EP samples and normal controls. Furthermore, machine learning techniques and the CIBERSORT algorithm were employed to extract immune characteristic genes specific to SP-EP patients, thereby enhancing the characterization of target genes. Next, we retrieved comprehensive information on patients diagnosed with SP-EP between 2000 and 2020 from the Surveillance, Epidemiology, and End Results Database (SEER). Using this data, we screened for predictive factors that have a significant impact on patient outcomes. A nomogram was constructed to visualize the predicted overall survival (OS) rates of these patients at 3, 5, and 8 years post-diagnosis. Finally, to assess the reliability and clinical utility of our predictive model, we evaluated it using various metrics including the consistency index (C-index), time-dependent receiver operating characteristic (ROC) curves, area under the curve (AUC), calibration curves, and decision curve analysis (DCA).

**Results:**

A total of 5,151 DEGs were identified between the SP-EP sample and the normal sample. Analysis of Gene Ontology (GO) and Kyoto Encyclopedia of Genes and Genomes (KEGG) pathways revealed that these DEGs were primarily involved in cellular processes, including cell cycle regulation and cell sensitivity mechanisms. Furthermore, immune infiltration analysis was utilized to identify the core gene *CELF4*. Regarding the survival rates of patients with SP-EP, the 3-year, 5-year, and 8-year survival rates were 72.5, 57.0, and 40.8%, respectively. Diagnostic age (*p* < 0.001), gender (*p* < 0.001), and surgical approach (*p* < 0.005) were identified as independent prognostic factors for OS. Additionally, a nomogram model was constructed based on these prognostic factors, demonstrating good consistency between predicted and actual results in the study’s validation process. Notably, the study also demonstrated that more extensive surgical resection could extend patients’ OS.

**Conclusion:**

Through bioinformatics analysis of microarray datasets, we identified *CELF4* as a central gene associated with immune infiltration among DEGs. Previous studies have demonstrated that *CELF4* may play a pivotal role in the pathogenesis of SP-EP. Furthermore, this study developed and validated a prognostic prediction model in the form of a nomogram utilizing the SEER database, enabling clinicians to accurately assess treatment risks and benefits, thereby enhancing personalized therapeutic strategies and prognosis predictions.

## 1 Introduction

SP-EP is a rare primary tumor of the central nervous system, typically arising from ependymoma cells in the spinal cord’s central canal. Its incidence peaks in adults aged 40–45 years (1, 2). Based on the WHO grading system, SP-EP is categorized into grades I-III, reflecting differences in cell heterogeneity and proliferative activity. Notably, the 2022 WHO diagnostic criteria for ependymoma saw mucinous papillary ependymoma elevated to grade II, with the anaplastic subtype no longer classified. Instead, specific subtypes are described histopathologically (3, 4). Clinical manifestations of SP-EP vary depending on tumor location, size, and growth rate, causing significant physical and mental distress to patients (5, 6). Surgical resection remains the primary treatment aiming for maximum safety. Guidelines recommend adjuvant radiotherapy (RT) for grade II primary SP-EP and all grade III cases post-surgery. However, the use of radiotherapy is controversial, and optimal dosing and prognostic benefits remain undetermined (7, 8). Systemic chemotherapy’s role in treating SP-EP is limited, with minimal lasting efficacy. Its impact on progression-free survival is also restricted. Therefore, chemotherapy is typically reserved as an adjuvant for recurrent cases where resection or radiotherapy is not feasible (9, 10).

Bioinformatics analysis, a powerful tool, uncovers potential molecular markers of disease by comparing gene expression patterns between patients and healthy controls (11). Nowadays, in-depth transcriptome bioinformatics analysis offers a fresh perspective in searching for diagnostic markers, prognostic indicators, and therapeutic targets. Nomogram, as a tool for comprehensive analysis and visual representation of prognostic risk factors, enable more accurate risk quantification. Notably, nomogram have been extensively utilized in prognostic assessments of intracranial mass lesions, including meningiomas, gliomas, and central lymphoma (12–14). However, due to limited data availability, there is currently no clinical prediction model tailored for SP-EP in practical application. Therefore, developing a novel model for this patient group is imperative. The GEO database^1^ serves as a widely accessed gene sequencing resource, enabling us to retrieve SP-EP-specific genetic information. Furthermore, the SEER database^2^ represents a reliable and extensive online platform for collecting cancer statistics in the US population.

## 2 Materials and methods

### 2.1 Data collection and analysis

In this study, RNA sequencing data from 14 SP-EP patients were retrieved from two chips in the GEO database: GSE66354 and GSE50161. To eliminate potential batch effects, the Combat method was applied to preprocess all RNA seq data. Subsequently, the annotation library “hgu133plus2.db” was utilized to map probe sets to their respective gene symbol identifiers. Probe sets annotated to the same gene symbol identifier were then aggregated using their average values (15). For the GSE54934 dataset, the “limma” package in R software was employed to identify DEGs between tumor samples and normal samples (16). DEGs were selected using a cutoff criterion of |log2FC| > 1 and an adjusted *p*-value (*P* adj) < 0.05 (15, 17). This approach allowed us to filter out significant DEGs for further analysis.

### 2.2 Functional enrichment analysis

To identify DEGs between two subgroups and understand their functional clustering, the “clusterProfiler” package in R software is utilized. This package performs statistical analysis and visualization of gene set functional clustering, providing insights into the biological roles of the identified DEGs (18). Furthermore, KEGG pathway enrichment analysis is conducted using the “clusterProfiler” package to investigate the main metabolic and signaling pathways associated with the DEGs (19). This analysis helps to identify the key biological processes and interactions underlying the observed gene expression differences. Gene Ontology (GO) is a comprehensive ontology in bioinformatics, encompassing three core domains: biological processes (BP), cellular components (CC), and molecular functions (MF) (20). Through KEGG pathway annotation and analysis of DEGs, the primary metabolic and signaling pathways associated with these genes can be identified (21). A significance threshold of *p* < 0.05 is adopted for enrichment analysis.

### 2.3 Utilizing machine learning techniques to identify disease-specific characteristic genes

To search for disease characteristic genes among differentially expressed genes, we initially employed the LASSO regression method. This approach utilized the “glmnet” package in R software to filter the expression levels of differentially expressed genes. Cross-validation was then conducted to identify the gene with the minimum error as the disease characteristic gene. Furthermore, we also screened disease characteristic genes using the SVM-RFE method. This involved filtering differential gene expression through the “e1071,” “kernlab,” and “caret” packages in R software. Similarly, cross-validation was employed to determine the gene with the lowest error as the disease characteristic gene. Finally, we intersected the disease characteristic genes identified by both methods and generated a VENN diagram to select the final set of disease characteristic genes.

### 2.4 Immune infiltration analysis of chips: a methodological perspective

Utilizing R software (version 4.3.1), along with the CIBERSORT algorithm, we conducted an analysis of the previously obtained and corrected gene expression matrix from the joint chip. This analysis aimed to identify genes with a significance level of *p* < 0.05 and to assess the proportion of 22 distinct species present in each sample. Furthermore, the CIBERSORT algorithm was employed to quantify the proportion of infiltrating immune cells. Additionally, the “limma” package within R software was leveraged to compare the ratios between high-risk and low-risk groups.

### 2.5 Research design and data collection

**Figure 1:**
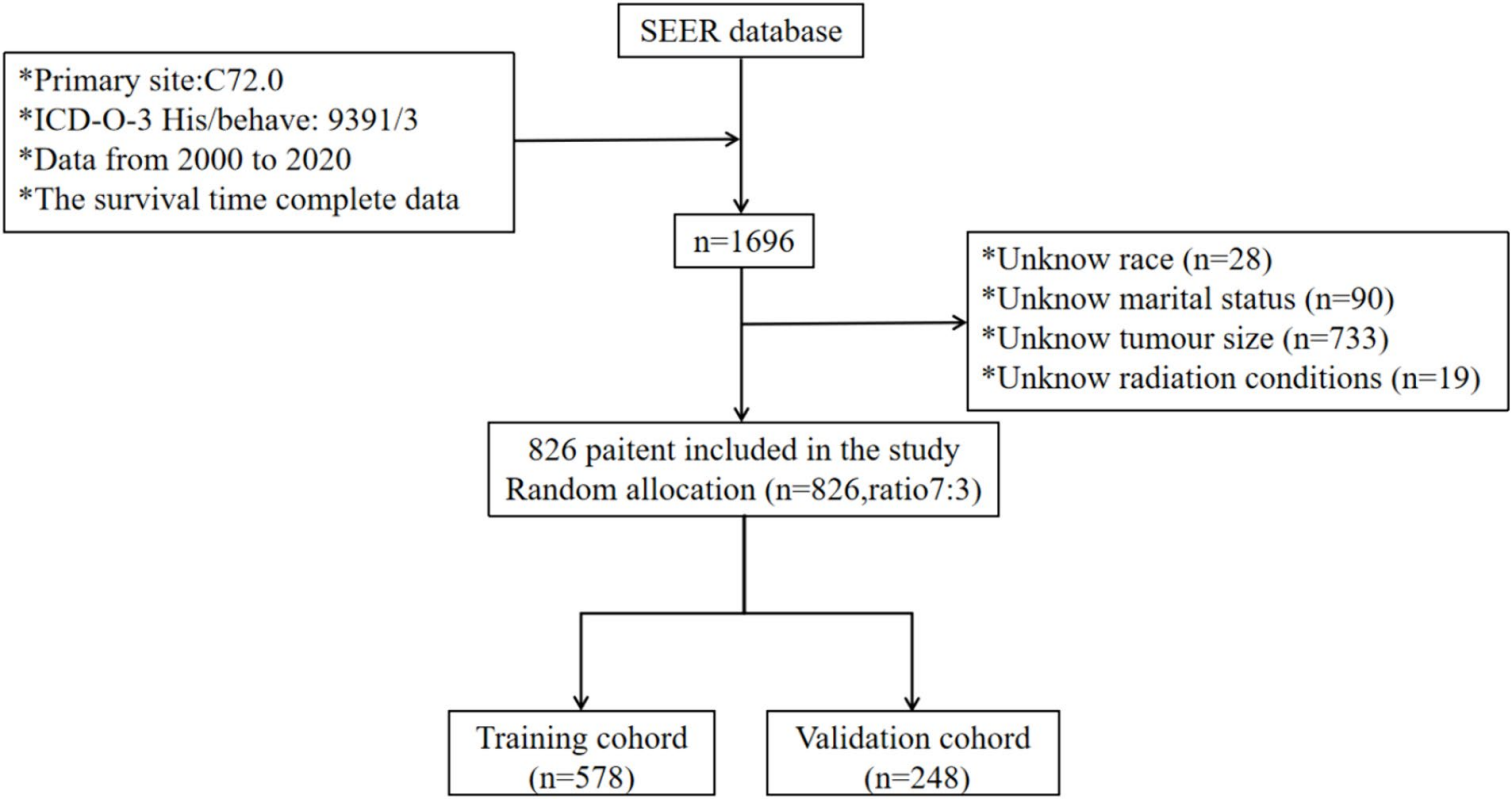
Participant inclusion and exclusion flowchart. SEER, Surveillance, Epidemiology, and End Result Program; ICD-0-3, International Classification of Disease for Oncology, Third Edition.

The SEER research data is accessible for public utilization by registered users, and the committee has exempted the necessity for informed consent, thereby eliminating the requirement for patient consent (license number: 13950, November 2021). Leveraging the SEER database, which was released in April 2024, we identified 1,696 patients diagnosed with SP-EP. The data retrieval process was facilitated by the SEER*Stat software, specifically version 8.4.3. To pinpoint patients with SP-EP, we utilized the primary tumor site code (C72.0), as stipulated in the third edition of the International Classification of Diseases for Oncology. Additionally, the histological code (ICD-O-3:9391/3) specific to ependymoma was employed for further classification. During our selection process, we excluded patients with the following characteristics: (1) unknown race and marital status, (2) unspecified tumor size (codes: 000/990/991/994/995/999), and (4) undetermined radiation therapy status. Consequently, as illustrated in Figure 1, 826 cases were ultimately included in our subsequent research and were randomly allocated to the training and validation sets in a 7:3 ratio.

### 2.6 Variable selection and research design

Clinical information is derived from the SEER database, encompassing various patient characteristics such as age at diagnosis, gender (male or female), racial categories (white, black, or other), marital status (married, unmarried, or unknown), tumor dimensions, tumor count (single or multiple), surgical intervention details, radiation therapy status (received or not received), and chemotherapy administration (administered or not administered). The X-tile program is utilized to categorize patients based on age into two groups: those aged ≤64 years and those >64 years. Tumor size is categorized using the median value as the cutoff. Surgical resection extents are classified into three categories: non-surgical resection, biopsy/STR, and total resection. The primary endpoint for measurement is OS. The conclusion of the follow-up period is set as December 31, 2020.

### 2.7 Construction and validation of nomograms

Using the cph() function in the RMS package of R language software, we constructed a predictive model that relies on independent prognostic factors to forecast the 1-year, 2-year, and 3-year OS rates among patients with SP-EP. Subsequently, we employed the plot() functions to generate corresponding survival prediction nomograms and visualize the prediction model. To identify independent prognostic variables, univariate and multivariate Cox regression analysis was conducted on the training dataset. The significant variables derived from this analysis were then utilized to develop nomograms for predicting the OS of SP-EP. To assess the performance of the nomograms, we utilized various evaluation methods. Specifically, the calibration curve was used to demonstrate the accuracy of nomogram predictions. Additionally, the time-dependent ROC curve and AUC were calculated to evaluate the nomograms’ ability to discriminate between different patient groups over time. Finally, to confirm the robustness of our findings, the nomograms were tested on the validation dataset and reanalyzed accordingly.

### 2.8 Clinical correlation

Perform DCA to evaluate the clinical utility of nomograms for practical clinical applications. The optimal critical value for each patient’s risk score is determined using the ROC curve. After calculating the risk score, patients in the training and validation cohorts are classified into high-risk and low-risk groups. To evaluate survival differences, we employed K-M survival curves to analyze OS differences between these groups. Additionally, we examined the influence of various surgical conditions on survival times between high-risk and low-risk patients.

### 2.9 Statistical analysis

We apply *χ*^2^. We verify the clinical variables between the training set and validation set and conduct univariate and multivariate Cox proportional hazards regression analysis to identify independent predictors of survival specifically within the training set. The consistency index (C-index) serves as a metric to assess the authenticity and reliability of the model represented by the nomogram. A calibration chart is constructed to assess the agreement between predicted and observed values. DCA is used to analyze the effectiveness of commonly used nomograms and prognostic indicators in clinical practice. Kaplan Meier method and logarithmic rank test are utilized for survival analysis. All statistical analyses were conducted using R software (version 4.3.1). The R packages used in this study include “rms,” “survival,” “surveyor,” and “ggDCA.” All statistical significance in this study was determined using a *p* < 0.05.

## 3 Results

### 3.1 RNA-seq gene differential analysis

**Figure 2:**
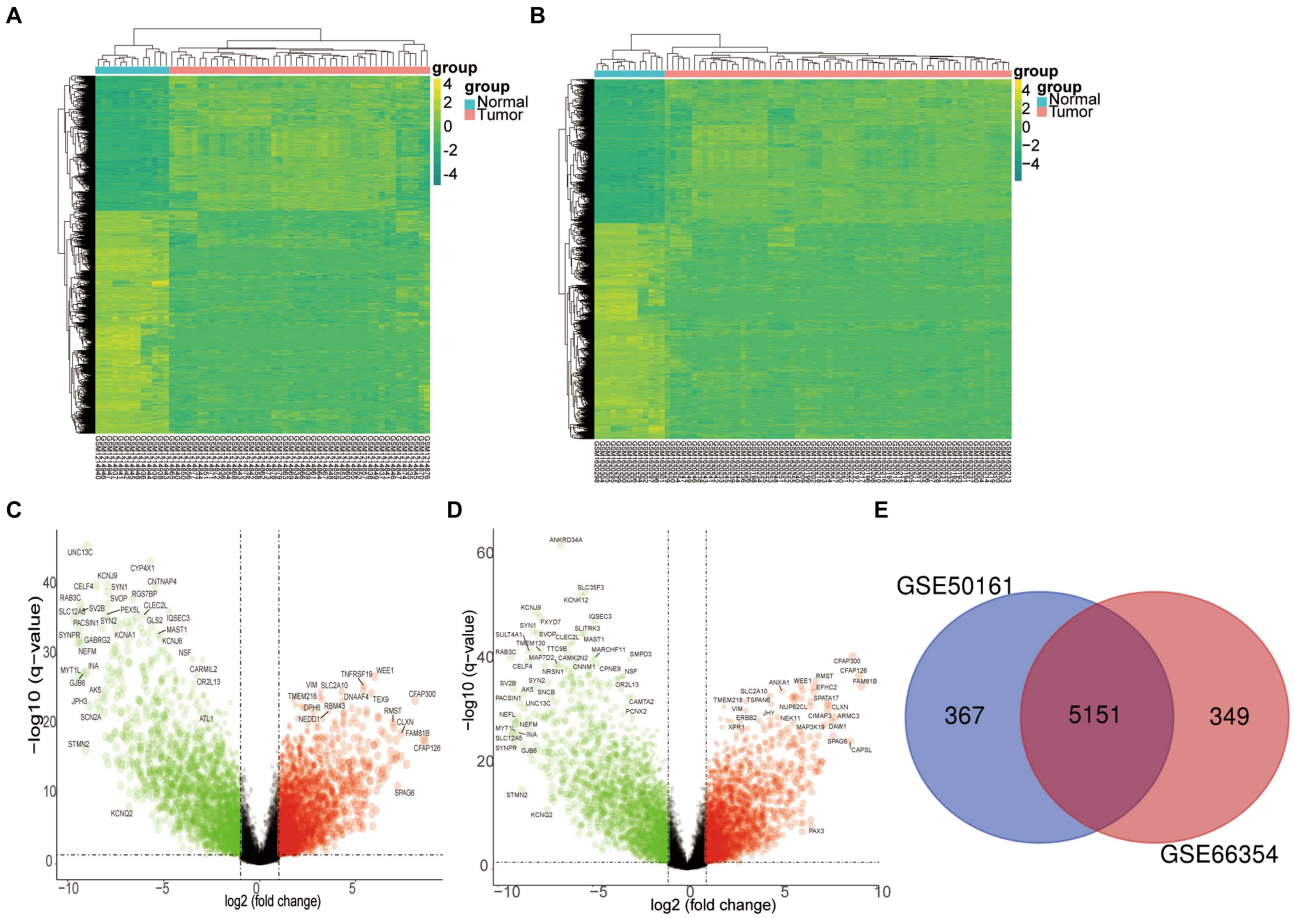
Differentially expressed genes. **(A)** The heatmap of DEGs in GSE50161 was generated using R software, with expression profiles above the mean depicted in yellow and those below in green. **(B)** Similarly, a heatmap for DEGs in GSE66354 was created through R software. **(C)** A volcano plot was constructed to visualize the differentially expressed genes in GSE50161, log FC: log2 fold change. **(D)** A volcano plot was also generated for the differentially expressed genes in GSE66354. **(E)** A Venn diagram illustrates the intersection of DEGs between GSE50161 and GSE66354 databases.

To delve into the pathogenic core genes of SP-EP, we first retrieved mRNA expression profiles of SP-EP and normal tissues from GEO (GSE50161 and GSE66354). Subsequently, we filtered and identified DEGs for comparison with normal tissues. Our analysis revealed a total of 5,151 DEGs, of which 2,679 genes were upregulated (log2 FC > 1) and 2,472 genes were downregulated (log2 FC < −1). This finding is illustrated in Figure 2.

### 3.2 Functional enrichment analysis

**Figure 3:**
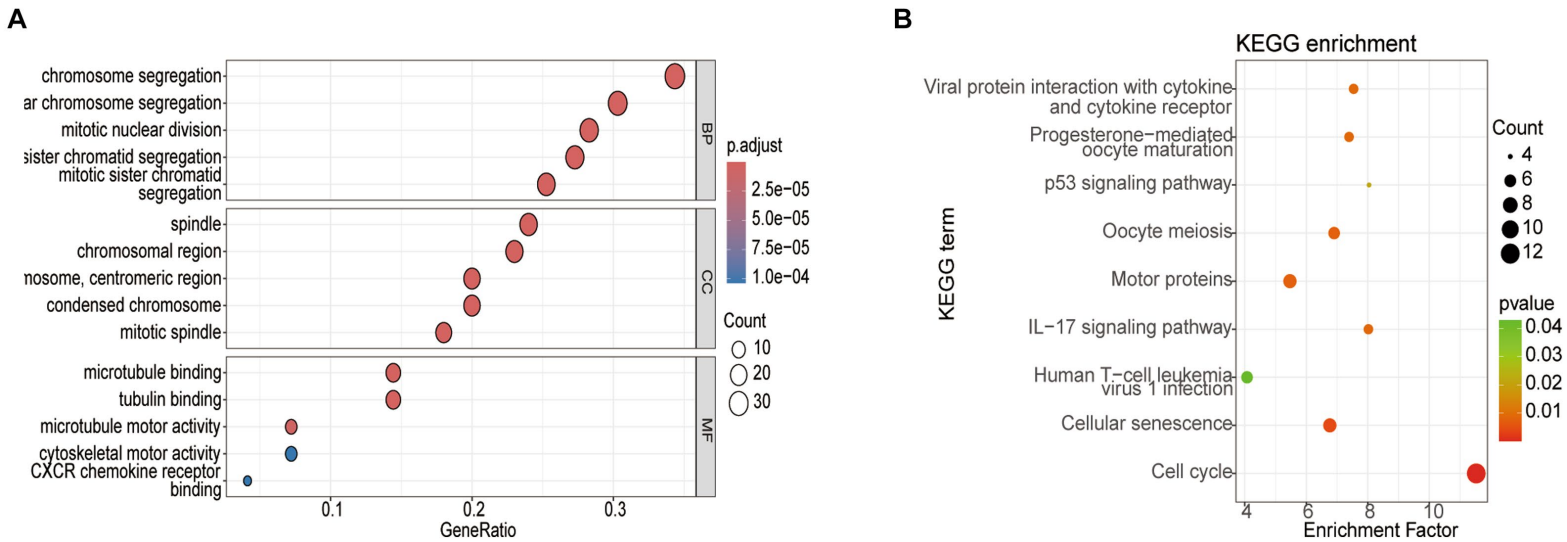
Analysis of the top 5 enriched GO and top 10 KEGG pathways in DEGs. **(A)** GO enrichment analysis. **(B)** KEGG enrichment pathway analysis. Node size represents the proportion of genes; The node color represents the *p*-value.

To delve deeper into the functions of the 5,151 DEGs in SP-EP, statistical analysis and visualization of their functional clustering were conducted using the “clusterprofile” package in R software. Table 1 presents the top 5 GO items of DEGs, sorted by *p*-values. From the BP analysis, it emerged that these DEGs primarily participate in processes such as chromosome segregation, nuclear chromosome segregation, sister chromatid segregation, mitotic sister chromatid segregation, and mitotic nuclear division. In the CC analysis, they were significantly associated with spindle, mitotic spindle, chromosome centromeric region, chromosomal region, and condensed chromosome. Furthermore, the MF analysis revealed that the DEGs are primarily involved in microtubule binding, tubulin binding, microtubule motor activity, cytoskeletal motor activity, and CXCR chemokine receptor binding. To gain additional insights into the crucial pathways of these DEGs, we conducted a KEGG pathway analysis. The results, depicted in Figure 3 and Table 2, revealed that the top 10 enriched KEGG pathways, ranked by *p*-values, primarily encompassed Cell cycle, Cellular senescence, Oocyte meiosis, Motor proteins, IL-17 signaling pathway, Viral protein interaction with cytokine and cytokine receptor, Progesterone-mediated oocyte maturation, p53 signaling pathway, and Human T-cell leukemia virus 1 infection.

**Table 1:** GO enrichment analysis of DEGs in SP-EP.

Category	Term	Count	Gene ratio	*p*-value
BP	Chromosome segregation	34	34/99	2.43E-31
BP	Nuclear chromosome segregation	30	30/99	6.47E-30
BP	Sister chromatid segregation	27	27/99	1.56E-29
BP	Mitotic sister chromatid segregation	25	25/99	9.75E-29
BP	Mitotic nuclear division	28	28/99	1.23E-28
CC	Spindle	24	24/100	1.07E-18
CC	Mitotic spindle	18	18/100	1.68E-18
CC	Chromosome, centromeric region	20	20/100	1.85E-18
CC	Chromosomal region	23	23/100	2.91E-18
CC	Condensed chromosome	20	20/100	9.95E-18
MF	Microtubule binding	14	14/97	1.56E-10
MF	Tubulin binding	14	14/97	1.09E-08
MF	Microtubule motor activity	7	7/97	6.54E-08
MF	Cytoskeletal motor activity	7	7/97	2.03E-06
MF	CXCR chemokine receptor binding	4	4/97	2.06E-06

**Table 2:** KEGG enrichment analysis of DEGs in SP-EP.

Category	Term	Count	Gene ratio	*p*-value
hsa04110	Cell cycle	12	12/58	3.21E-10
hsa04218	Cellular senescence	7	7/58	7.04E-05
hsa04114	Oocyte meiosis	6	6/58	0.000215
hsa04814	Motor proteins	7	7/58	0.000265
hsa04657	IL-17 signaling pathway	5	5/58	0.000377
hsa04061	Viral protein interaction with cytokine and cytokine receptor	5	5/58	0.000501
hsa04914	Progesterone-mediated oocyte maturation	5	5/58	0.000549
hsa04115	p53 signaling pathway	4	4/58	0.001491
hsa05166	Human T-cell leukemia virus 1 infection	6	6/58	0.003364

### 3.3 Machine learning method for obtaining disease characteristic genes

**Figure 4:**
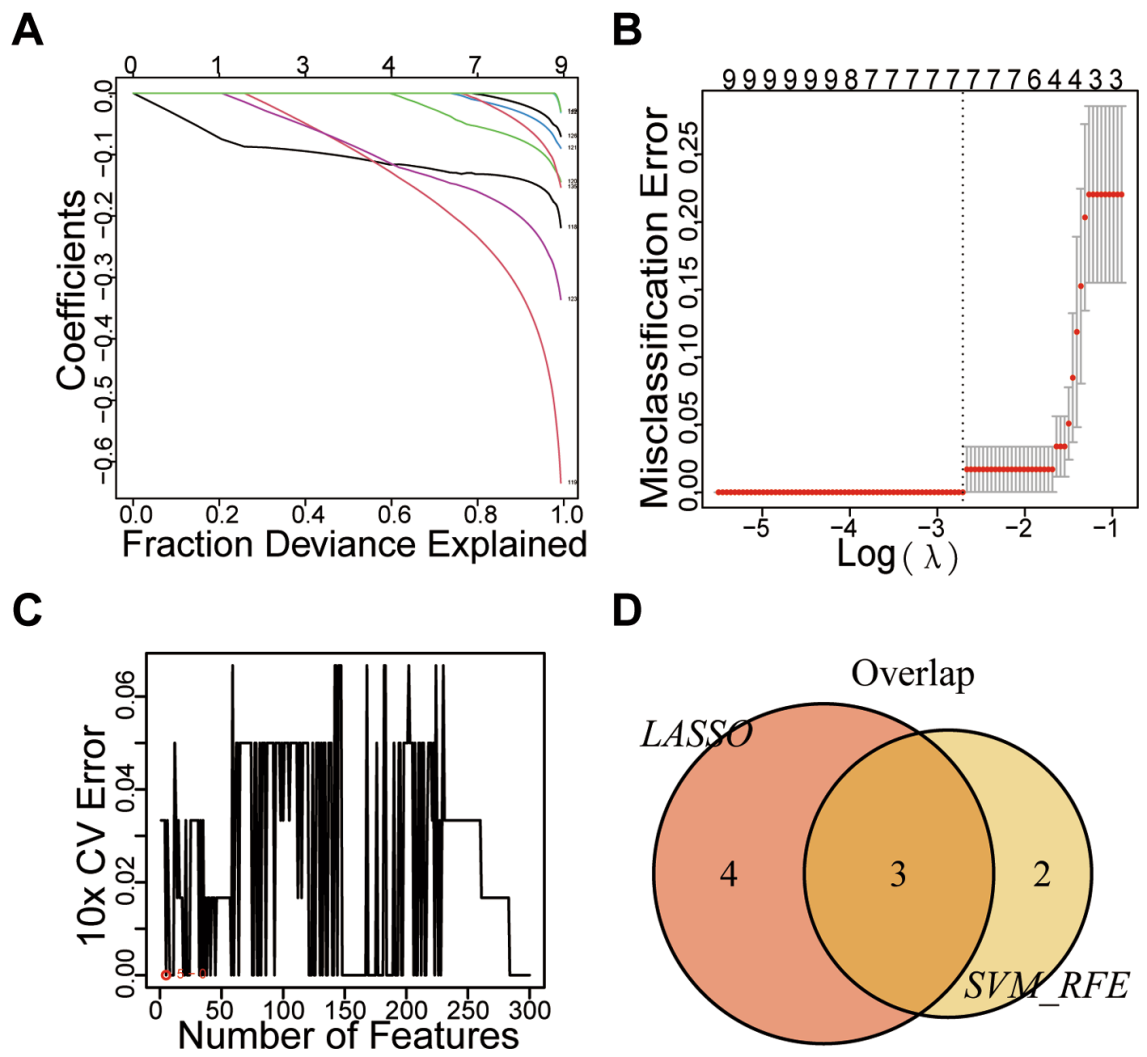
Mechanical Learning Method for Identifying Disease-Specific Genes. **(A)** LASSO Coefficient Curve Depicting Prognostic DEGs. **(B)** Cross-Validation for Selecting Optimal Regularization Parameters (λ). **(C)** SVM-RFE Diagram. **(D)** VENN Diagram.

We applied LASSO regression to filter differential gene expression using the R software “glmnet” package. Seven genes with the lowest error values were selected as disease characteristic genes, and relevant visualization graphs were drawn (Figures 4A,B). The vertical axis represents the error size, the horizontal axis represents the number of genes, and gene errors were ranked from high too low to obtain *UNC13C*, *CNTNAP4*, *SYN1*, *CELF4*, *CYP4X1*, *SEC14L5*, and *CLEC2L*. Furthermore, we utilized the SVM-RFE method to screen disease characteristic genes, employing the R software packages “e1071,” “kernlab,” and “caret” to filter differential gene expression. We employed cross-validation to identify the disease characteristic genes with minimal error and conducted visual analysis (Figure 4C). The vertical axis represents the error size, and the horizontal axis represents the number of genes. Thirty-one genes with minimal error expressions were identified. The intersection of the disease characteristic genes obtained from both methods was taken, and a Venn diagram was drawn (Figure 4D) to obtain *SYN1*, *CELF4*, and *CYP4X1*.

### 3.4 Immune infiltration analysis

**Figure 5:**
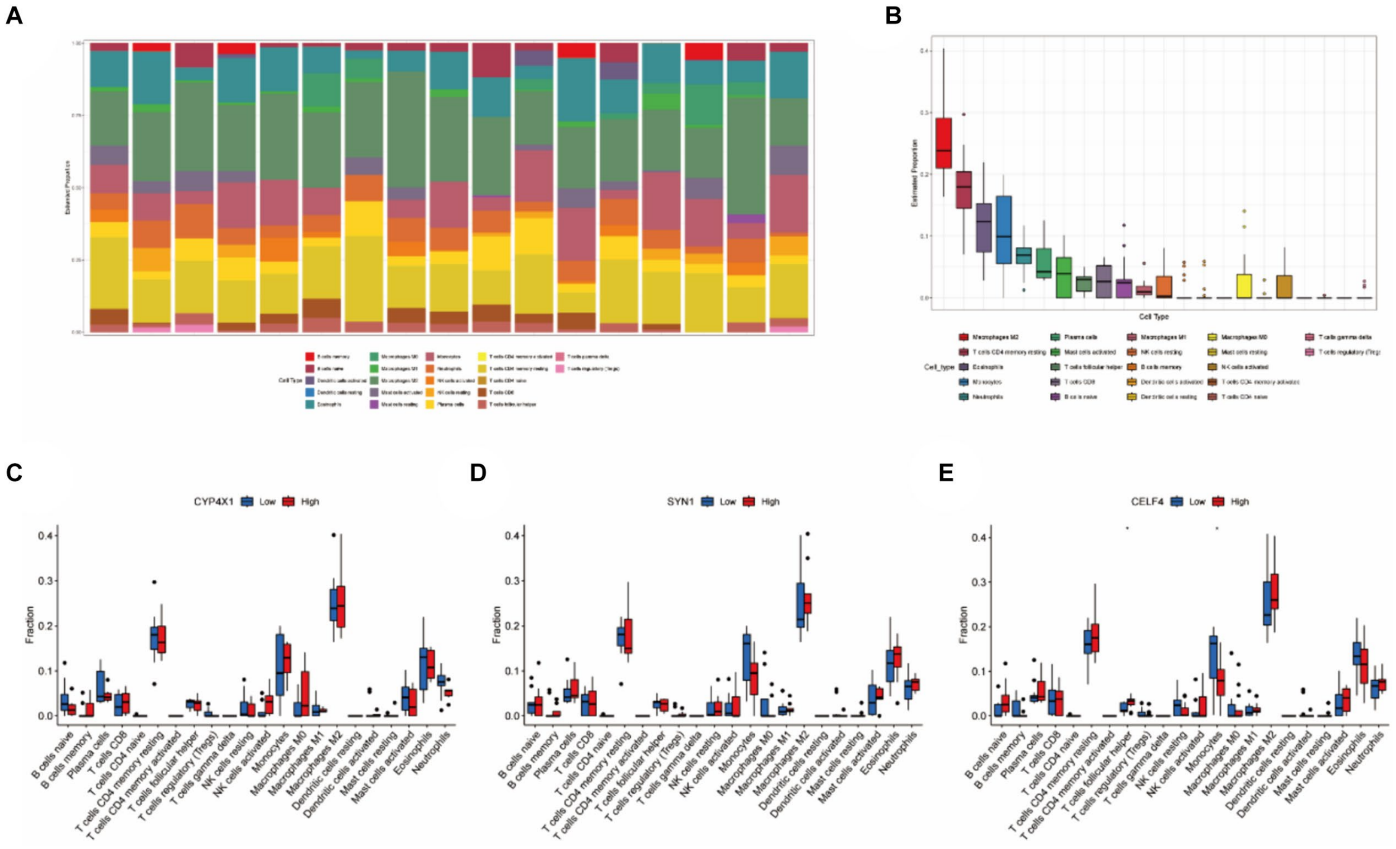
Immune infiltration analysis based on 22 immune related gene sets. **(A)** Representing 22 subtypes of immune cells, each bar chart abundance represents the proportion of immune cells in each sample, and different colors represent each subtype. **(B)** Differences in the proportion of 22 subtypes of immune cells. **(C–E)** Comparative analysis of the proportion of immune infiltrating cells between the high-risk and low-risk groups of *CELF4*, *SYN1*, and *CYP4X1* (^*^p < 0.05).

Based on 22 immune-related gene sets, we conducted immune infiltration analysis, which revealed the subtypes of immune cells, their corresponding abundances, and variations in the proportions of various immune cells across tumor samples. As depicted in Figures 5A,B, the proportions of Macrophages M2, CD4+ memory resting T cells, Eosinophils, Monocytes, and Neutrophils were comparatively high in SP-EP tumor tissue, whereas the proportions of other immune cell types were relatively low. To further investigate the association between three key genes and immune cell infiltration, patients were stratified into high-and low-risk groups. The results are presented in Figures 5C–E. Notably, a significant difference was observed in the proportions of follicular helper T cells and Monocytes between the two groups in the context of *CELF4* (*p* < 0.05). However, no significant differences were detected for *SYN1* and *CYP4X1* (*p* > 0.05).

### 3.5 Clinical characteristics of patients

Our study encompassed 826 patients diagnosed with SP-EP in the SEER database spanning the years 2000–2020. Of these patients, the training set comprised 578 individuals (70%) and the validation set consisted of 248 individuals (30%). In terms of age distribution, the majority of patients (87.3%) fell within the young age bracket of ≤64 years, with 12.7% belonging to the elderly group (>64 years). Regarding gender, 47.2% were male and 52.8% were female. Notably, young women under 64 years of age constituted the primary affected group, accounting for 45.9 and 46.5% in the training and validation sets, respectively. In terms of tumor characteristics, male patients exhibited an average tumor size of 34.6 mm, whereas female patients had an average tumor size of 27.9 mm. When it came to treatment options, surgical intervention was the most preferred method, with 78.3% of patients opting for it. Radiotherapy followed as the second most common choice, accounting for 16.3% of patients. Chemical drug treatment, however, was chosen by only a minuscule proportion of 0.4%, and none of these patients underwent either surgery or radiation therapy. It is important to mention that sequential variables related to surgery, radiotherapy, and chemotherapy are not recorded in the SEER database. Similarly, detailed information about the drugs used in chemotherapy is also unavailable. For a comprehensive overview of the clinical data, please refer to Table 3.

**Table 3:** Patient characteristics and socio-demographic.

Characteristics	Training cohort (*n* = 578), *n* (%)	Validation cohort (*n* = 248), *n* (%)	*p*-value
**Age (*n*%)**			
≤64 years	501 (86.7%)	220 (88.7%)	721 (87.3%)
>64 years	77 (13.3%)	28 (11.3%)	105 (12.7%)
**Sex (*n*%)**			
Male	272 (47.1%)	118 (47.6%)	390 (47.2%)
Female	306 (52.9%)	130 (52.4%)	436 (52.8%)
**Race (*n*%)**			
White	488 (84.4%)	219 (88.3%)	707 (85.6%)
Black	52 (9.0%)	11 (4.4%)	63 (7.6%)
Other	38 (6.6%)	18 (7.3%)	56 (6.8%)
**Marital status (*n*%)**			
Single	151 (26.1%)	62 (25.0%)	213 (25.8%)
Married	343 (59.3%)	158 (63.7%)	501 (60.7%)
Others	84 (14.5%)	28 (11.3%)	112 (13.6%)
**Size (*n*%)**			
<30 mm	353 (61.1%)	160 (64.5%)	513 (62.1%)
≥30 mm	225 (38.9%)	88 (35.5%)	313 (37.9%)
**Number (*n*%)**			
Single	494 (85.5%)	217 (87.5%)	711 (86.1%)
Multiple	84 (14.5%)	31 (12.5%)	115 (13.9%)
**Surgery (*n*%)**			
GTR	163 (28.2%)	86 (34.7%)	249 (30.1%)
Biopsy/STR	288 (49.8%)	110 (44.4%)	398 (48.2%)
NO surgery	127 (22.0%)	52 (21.0%)	179 (21.7%)
**Radiation (*n*%)**			
Yes	99 (17.1%)	36 (14.5%)	135 (16.3%)
No	479 (82.9%)	212 (85.5%)	691 (83.7%)
**Chemotherapy (*n*%)**			
Yes	2 (0.3%)	1 (0.4%)	3 (0.4%)
No	576 (99.7%)	247 (99.6%)	823 (99.6%)

GTR, total resection; RT, radiotherapy; STR, subtotal resection.

### 3.6 Variable selection

**Figure 6:**
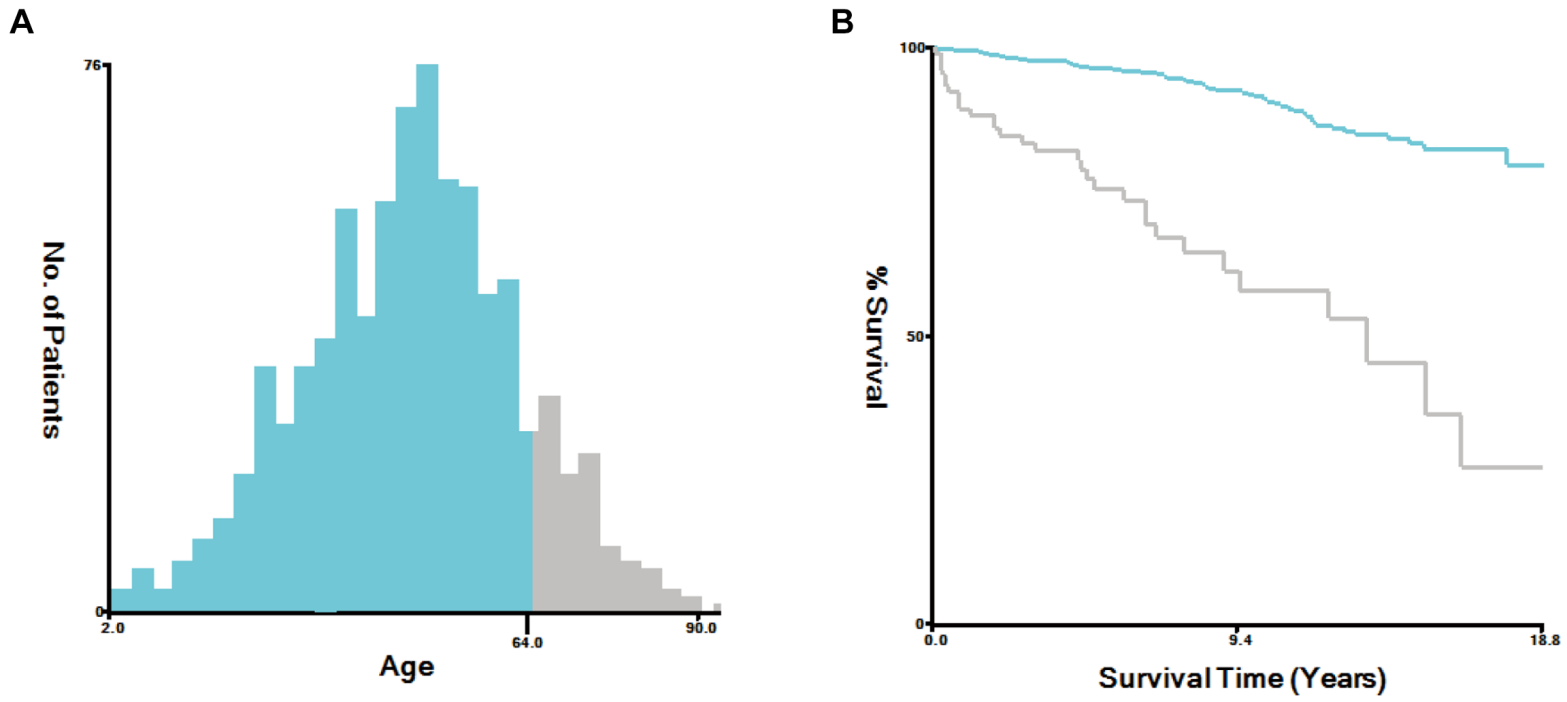
Sing X-Tile software to determine the optimal cutoff values for age. **(A)** Age-Survival Histogram. **(B)** Age-Survival Kaplan Meier diagram.

In this study, the optimal cutoff value for continuous variables was determined using X-Tile software (version 3.6.1). The patient’s age was categorized into two groups: ≤ 64 years old and > 64 years old, as depicted in Figure 6. To assess the interaction among various covariates, relevant factors with *p* < 0.05 in both univariate and multivariate Cox proportional risk models were combined to identify independent prognostic factors. The findings revealed that age (*p* < 0.001), gender (*p* < 0.001), and surgical method (*p* < 0.005) served as independent predictors of prognosis, as summarized in Table 4. Notably, younger age, female gender, and complete surgical resection were factors that significantly contributed to improved overall survival (OS) in patients with SP-EP.

**Table 4:** Univariate and multivariate analyses of characteristics for predicting overall survival (OS) in patients with SP-EP.

Variables	Univariate			Multivariate		
	HR	95%CI	*p*-value	HR	95%CI	*p*-value
**Age (years)**						
≤64	Ref.			Ref.		
>64	6.1	3.9–9.5	*p* < 0.001*	5.2633	2.6847–8.6682	*p* < 0.001*
**Sex**						
Male	Ref.			Ref.		
Female	0.58	0.38–0.89	*p* < 0.001*	0.6418	0.3697–1.0803	*p* < 0.001*
**Race**						
White	Ref.			Ref.		
Black	−0.04	0.52–2.5	*p* = 0.95	1.6197	0.6102–4.2958	*p* = 0.333
Others	−0.04	0.39–2.4		1.6474	0.4935–5.4941	*p* = 0.417
**Marital status**						
Single	Ref.			Ref.		
Married	0.44	0.51–1.4	*p* = 0.089	0.4858	0.3800–1.4747	*p* = 0.552
Divorced/separated/widowed	0.44	0.83–2.9		1.0883	0.4880–2.4275	*p* = 0.803
**Size (mm)**						
≤30	Ref.			Ref.		
>30	1.23	0.64–1.5	*p* = 0.94	1.2460	0.7203–2.1572	*p* = 0.431
**Tumor number**						
Single	Ref.			Ref.		
Multiple	3.7	2.4–5.7	*p* = 0.013*	3.3463	1.8936–5.9127	*p* = 0.093
**Surgery**						
GTR	Ref.			Ref.		
Biopsy/STR	0.84	0.82–2.5		1.3168	0.6426–2.6946	*p* = 0.452
NO surgery	0.84	1.4–3.9	*p* = 0.0046*	9.1490	1.3045–4.7786	*p* < 0.005*
**Radiation**						
Yes	Ref.			Ref.		
No	0.73	0.44–1.2	*p* = 0.22	7.6595	0.4126–1.422	*p* = 0.398
**Chemotherapy**						
Yes	Ref.			Ref.		
No	0.2	0.028–1.5	*p* = 0.11	1.3764	0.0000-Inf	*p* = 0.996

GTR, total resection; RT, radiotherapy; STR: subtotal resection. *p < 0.05; HR, hazard ratio; CI, confidence interval.

### 3.7 Nomogram validation

**Figure 7:**
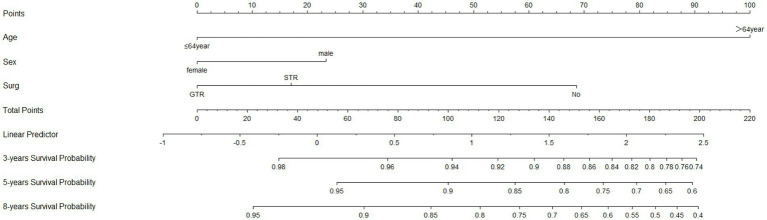
The nomograms to predict OS at 3-, 5-, and 8-year with SP-EP. SEER, Surveillance, Epidemiology, and End Result Program; OS, overall survival.

**Figure 8:**
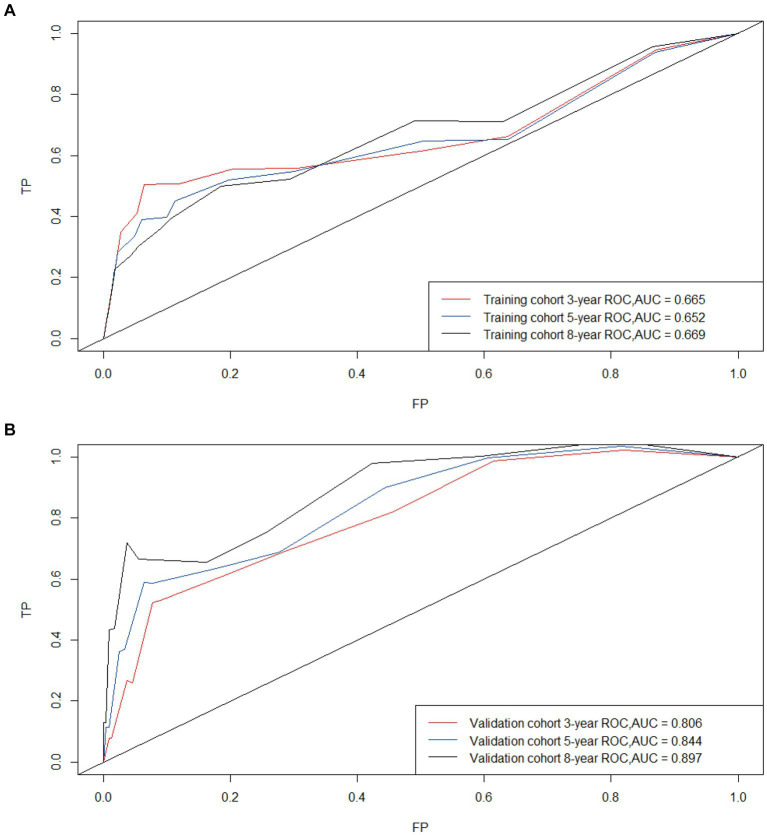
Time-dependent curves (ROC) of the nomogram for 3-, 5-, and 8-year predictions; AUC for predicting OS in the training cohort **(A)** and validation cohort **(B)**.

**Figure 9:**
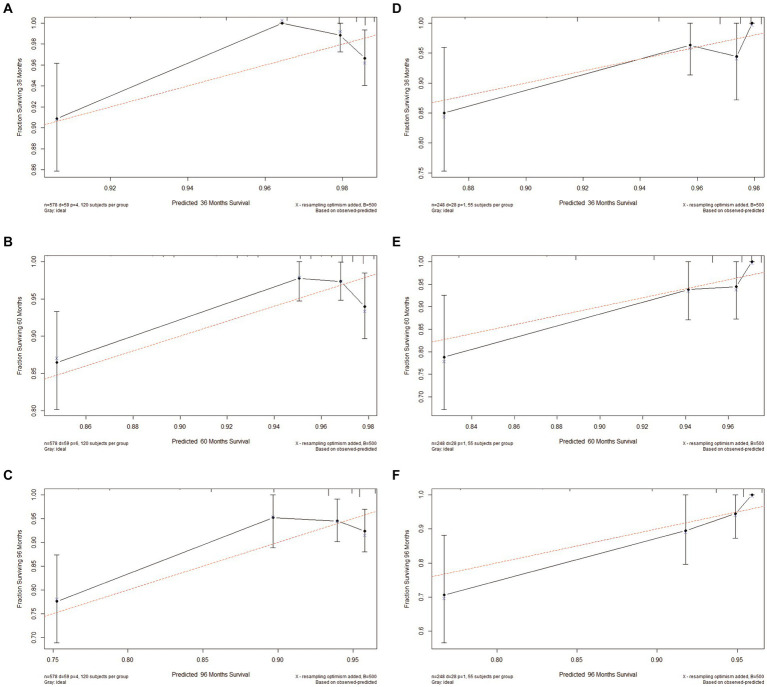
Calibration plots of 3-, 5-, and 8-year OS for patients with intramedullary SP-EP. **(A–C)** Calibration plots of 3-, 5-, and 8-year OS in the training cohort. **(D–F)** Calibration plots of 3-, 5-, and 8-year OS in the validation cohort.

**Figure 10:**
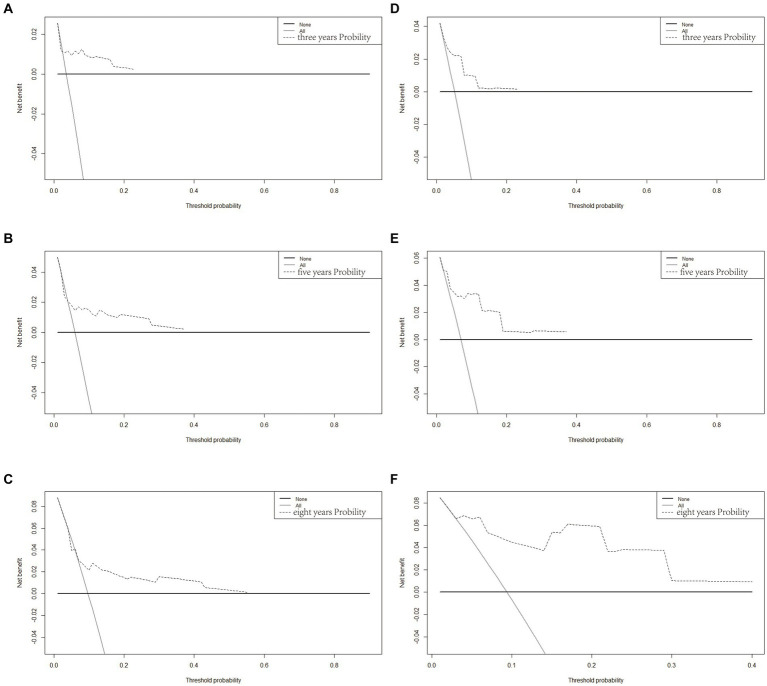
Decision curve analysis of the OS-associated nomograms. DCA curves of 3-, 5-, and 8-year OS in the training cohort **(A–C)** and validation cohort **(D–F)**.

Based on Cox univariate/multivariate regression analysis, we constructed prognostic models for 3-year, 5-year, and 8-year overall survival (OS) in SP-EP patients. The results, visualized in Figure 7 as nomogram, reveal that age is the most significant prognostic factor, followed by surgical methods and gender differences. Each factor level is assigned a grade score, enabling visual calculation through the nomogram. Summing the scores across all factors provides the corresponding OS value. In both the training and validation sets, the C-index of the OS prediction model is 0.741 and 0.747, respectively (Table 5). Additionally, the AUC values for 3, 5, and 8 years indicate good discriminability of the prediction model (Figure 8). To assess the calibration level of the OS prediction models, we employed calibration curve graphs. Both the modeling and validation groups demonstrate a high overlap between the calibration curve and the standard line (Figure 9), indicating a strong correlation between the predicted and observed survival rates. Furthermore, we evaluated the clinical applicability of the prediction model using DCA (Figure 10). The results demonstrate a wide threshold probability range and a high net benefit for predicting 3-year, 5-year, and 8-year survival rates in SP-EP patients.

**Table 5:** C-index for training and validation sets in OS nomograms.

	Training set		Validation set	
	C-index	95%CI	C-index	95%CI
OS	0.741		0.747	

C-index, concordance index; OS, overall survival; CI, confidence interval.

## 4 Discussion

In this study, we retrieved spinal meningioma data from the GEO database and conducted a comprehensive analysis of the genetic profiles of affected patients. Differential gene analysis revealed a significant enrichment of genes primarily associated with chromosome segregation, nuclear chromosome segregation, and sister chromatid segregation. These genes are intricately involved in cellular processes such as cell cycle regulation, cellular sensitivity mechanisms, meiotic events in oocytes, motor protein functions, and the IL-17 signaling pathway—all of which are closely linked to immunological functions. Subsequently, we identified three core genes: *CELF4*, *SYN1*, and *CYP4X1*. Notably, *CYP4X1* appears to play a pivotal role in the pathogenesis and immune infiltration associated with SP-EP.

*CELF4* (CUGBP Elav Like Family Member 4), an RNA-binding protein, plays diverse roles in cellular processes such as RNA splicing and mRNA stability. Its expression has been shown to influence the immune response within the tumor microenvironment. Specifically, studies suggest that *CELF4* might suppress the anti-tumor immune response by regulating immune checkpoint molecules (22, 23). Additionally, *CELF4* expression has been associated with poor prognosis in colorectal cancer, indicating its significance in the pathogenesis of this malignancy (24). Immune tumor infiltration is crucial in cancer development, metastasis, and immune escape, affecting patient prognosis (25–27).

*CELF4* is widely expressed, with high levels observed in the central nervous system (28). SP-EP, a rare tumor originating from ependymal cells, remains understudied regarding the role of *CELF4*. However, insights can be drawn from studies on *CELF4’*s function in other CNS tumors, such as glioblastoma, which suggest its involvement in tumor progression and aggressiveness (29–31). The exact mechanism underlying *CELF4*’s influence on tumor immune infiltration in SP-EP remains elusive. Based on findings in other cancers, it is hypothesized that *CELF4* may regulate the expression of genes involved in antigen presentation, cytokine signaling, and immune cell recruitment within the tumor microenvironment (22, 32). Future research is warranted to delve deeper into the specific role of *CELF4* in SP-EP and its intricate interplay with the tumor immune response. Studies exploring *CELF4* expression levels in patients with SP-EP and their correlation with immune cell infiltration would be highly valuable. Additionally, functional studies aimed at elucidating the precise mechanisms by which *CELF4* regulates immune infiltration in this specific cancer type are urgently needed.

In addition, we conducted an extensive retrospective study focusing on the clinical data of SP-EP patients, thereby presenting the most recent evidence for their epidemiological analysis. Utilizing the SEER database, we developed clinical prognostic models that encompassed 3-year, 5-year, and 8-year survival probabilities by extracting potential prognostic factors specific to patients with SP-EP. Our findings indicate that age, gender, and surgical approach are potentially significant predictors of survival outcomes for SP-EP patients.

Consistent with previous findings by Boström et al. (33), age serves as a crucial factor influencing the prognosis of SP-EP patients. In cases where other tumor characteristics remain constant, the overall survival rate (OS) among older patients is significantly lower compared to younger patients, displaying a statistically significant difference (34). Through a combination of univariate/multivariate Cox regression analysis and the clinical prediction nomogram established, it becomes evident that an increase in age is inversely associated with patients’ overall survival time. This could be attributed to the higher incidence of postoperative long-term sequelae in older patients, including secondary hydrocephalus, neurological dysfunction, and nutritional and metabolic disorders (35, 36). Additionally, this trend may also be linked to age-related declines in immune function and gene repair capacity (9). Male gender emerges as a significant prognostic indicator for poorer outcomes in ependymoma, particularly among young boys under 15 years of age (37–40). According to population-based cancer registry data analyzed by Soon et al. (41), there is a notable trend indicating an improved survival rate among female patients. Notably, the median OS for female patients diagnosed with malignant ependymoma is significantly longer than that of males (262 months versus 196 months). However, the precise reasons underlying the overall longer survival time observed in female patients remain elusive.

Surgical resection has traditionally been regarded as the primary treatment approach for SP-EP. Our research aligns with this consensus, revealing that patients undergoing ependymoma surgery fare better, with the extent of resection serving as a significant prognostic factor (42, 43). Notably, across various studies, patients who undergo gross total resection (GTR) surgery exhibit superior outcomes compared to those undergoing subtotal resection (STR) surgery (37, 44). Our findings echo these previous observations, emphasizing the need to prioritize maximal tumor tissue removal while safeguarding neurological function to mitigate recurrence risks. Advancements in microsurgical techniques and endoscopic surgery have significantly enhanced the prognostic outlook for patients with SP-EP.

The utilization of postoperative radiotherapy (RT) in the management of ependymoma is gradually escalating (45). Nevertheless, our investigation reveals that adjuvant radiotherapy did not markedly enhance the overall survival (OS) of patients regardless of whether they underwent complete or incomplete resection surgery. Furthermore, certain studies concur that radiotherapy confers no notable advantage in terms of OS for ependymoma patients. In contrast to our findings, prior studies have advocated for high-dose adjuvant radiotherapy in patients undergoing subtotal resection (STR) (45, 46). However, it is noteworthy that our research may be constrained by the limitations inherent in the SEER database, which precludes us from obtaining detailed information regarding tumor radiation dose and radiation field.

Over the past decade, tumor prediction models have increasingly been adopted, with nomogram emerging as one of the preferred methods. In our study, we also employed nomogram to predict patient outcomes. Compared to older prediction models, nomogram exhibit greater accuracy and effectiveness in forecasting patient outcomes, thanks to their balanced consideration of various factors (47). Furthermore, our analysis revealed that nomogram can precisely predict patient survival rates. Leveraging this approach, we developed a risk classification system that stratifies patients into high, medium, and low-risk OS categories. This system serves as a valuable tool for guiding patient risk adaptation counseling and clinical treatment decisions. However, it’s worth noting that our study has several limitations. Firstly, being a retrospective cohort study, it is inevitably prone to potential selection bias, which typically renders such studies less robust than large randomized controlled trials. Secondly, our validation process was limited to internal data, and we eagerly anticipate external validation using other datasets in the future. Additionally, the SEER database lacks detailed information on chemotherapy usage, radiation methods, and specific radiation levels, thus hindering our ability to correlate delayed treatment effects with precise radiation doses/volumes or specific chemotherapy administrations.

In conclusion, we have successfully developed and internally validated a nomogram-based OS prediction model for patients with SP-EP. Despite its limitations, the model demonstrates acceptable accuracy and clinical applicability, offering medical professionals a practical tool for intuitive and personalized risk analysis in clinical practice.

## Data availability statement

Publicly available datasets were analyzed in this study. This data can be found at: SEER (https://seer.cancer.gov/) and GEO database (https://www.ncbi.nlm.nih.gov/geo/) with accession numbers: GSE66354 and GSE50161.

## Ethics statement

Ethical approval was not required for the study involving humans in accordance with the local legislation and institutional requirements. Written informed consent to participate in this study was not required from the participants or the participants’ legal guardians/next of kin in accordance with the national legislation and the institutional requirements.

## Author contributions

TF: Data curation, Formal analysis, Funding acquisition, Investigation, Methodology, Project administration, Supervision, Validation, Visualization, Writing – original draft. CM: Conceptualization, Data curation, Formal analysis, Investigation, Methodology, Project administration, Supervision, Validation, Visualization, Writing – original draft, Resources. ZC: Conceptualization, Data curation, Investigation, Methodology, Project administration, Resources, Supervision, Validation, Visualization, Writing – original draft. YH: Conceptualization, Methodology, Project administration, Validation, Visualization, Writing – original draft. HL: Investigation, Methodology, Project administration, Validation, Visualization, Writing – original draft, Conceptualization, Data curation, Formal analysis, Resources, Software. CW: Funding acquisition, Investigation, Project administration, Conceptualization, Data curation, Formal analysis, Methodology, Writing – review & editing. JL: Conceptualization, Data curation, Formal analysis, Funding acquisition, Investigation, Methodology, Project administration, Resources, Supervision, Validation, Visualization, Writing – review & editing, Software. SL: Conceptualization, Data curation, Investigation, Project administration, Resources, Software, Validation, Visualization, Writing – review & editing. FL: Conceptualization, Data curation, Funding acquisition, Investigation, Project administration, Resources, Visualization, Writing – review & editing, Formal analysis, Methodology, Supervision, Validation.

## Footnotes

^1^ https://www.ncbi.nlm.nih.gov/geo/

^2^ https://seer.cancer.gov/
